# Association between Gross Motor Competence and Physical Fitness in Chilean Children Aged 4 to 6 Years

**DOI:** 10.3390/children11050561

**Published:** 2024-05-08

**Authors:** Andrés Godoy-Cumillaf, Paola Fuentes-Merino, Frano Giakoni-Ramírez, Daniel Duclos-Bastías, José Bruneau-Chávez, Diego Vergara-Ampuero, Eugenio Merellano-Navarro

**Affiliations:** 1 Grupo de Investigación en Educación Física, Salud y Calidad de Vida (EFISAL), Facultad de Educación, Universidad Autónoma de Chile, Temuco 4780000, Chile; andres.godoy@uautonoma.cl (A.G.-C.); paola.fuentes@uautonoma.cl (P.F.-M.); 2 Faculty of Education and Social Sciences, Universidad Andres Bello, Las Condes, Santiago 7550000, Chile; frano.giakoni@unab.cl; 3 Escuela de Educación Física, Pontificia Universidad Católica de Valparaíso, Valparaíso 2340000, Chile; daniel.duclos@pucv.cl; 4 IGOID Research Group, Physical Activity and Sport Science Department, University of Castilla-La Mancha, 45071 Toledo, Spain; 5 Departamento de Educación Física, Deportes y Recreación, Universidad de la Frontera, Temuco 4811230, Chile; jose.bruneau@ufrontera.cl; 6 Escuela de Ciencias del Deporte y la Actividad Física, Facultad de Salud, Universidad Santo Tomás, Santiago 8370003, Chile; dvergara11@santotomas.cl; 7 Department of Physical Activity Sciences, Faculty of Education Sciences, Universidad Católica del Maule, Talca 3530000, Chile

**Keywords:** balance, aiming, catching, cardiorespiratory fitness, strength, speed/agility, preschool

## Abstract

The preschool period is considered critical for the development of motor competence, but as far as we know, no studies have investigated the association between motor competence and physical fitness in Chilean children. The aim of this study was to analyse the association between gross motor competence and physical fitness, controlling for possible confounding factors. A cross-sectional study was conducted with a sample of 144 preschool children (56.25% girls) with an average age of 5.3 years (4 to 6 years) from the Araucanía region, Chile. Motor competence was measured using the Children’s Movement Assessment Battery, 2nd Edition (MABC-2). Regarding physical fitness, the components of cardiorespiratory fitness, lower body muscle strength and speed/agility were evaluated using the Battery to Assess FITness in PREschool (PREFIT). Partial correlation models and analysis of variance (ANCOVA) were used to assess differences in physical fitness between motor competence categories, controlling for age and body mass index. The mean fitness scores for cardiorespiratory fitness, lower body muscle strength and speed/agility components were significantly higher in children with higher gross motor competence. In terms of effect size, large values were found for the lower body strength component in model 1 for boys and in model 2 for the total samples of girls and boys. The results of this study suggest that good levels of gross motor competence are associated with better physical fitness levels.

## 1. Introduction

Motor competence (MC) is defined as an individual’s ability to perform different motor actions efficiently and accurately [1]. It has also been proposed that MC refers to the degree of proficiency an individual demonstrates in the execution of a wide variety of motor skills [2]. Evidence suggests that if children do not develop adequate MC, they are unlikely to be able to successfully perform in a variety of physical activities throughout their lives [3,4].

MC consists of fine MC, which is involved in performing movements that require precision, such as drawing or writing, and is important in academic and social settings [5] and gross motor competence (GMC), which is involved in performing tasks such as running, jumping, or catching and is important as it provides the foundation for future motor development [5]. Low GMC scores are associated with poorer physical, psychological and social health and even affect cognitive performance [6].

On the other hand, health-related physical fitness (PF) is a measure that integrates most of the bodily functions involved in physical activity, encompassing different components that contribute to a person’s physical and mental well-being [7]. Its components are cardiorespiratory fitness, muscle strength, speed/agility, body composition and flexibility [7]. PF is considered an indicator of health in childhood, as optimal levels are associated with improved cardiovascular health, reduced total adiposity, reduced metabolic risk, increased bone mineral density and improved range of motion and coordination [8,9,10,11]. Studies evaluating PF in Chilean preschoolers are scarce, and most are conducted with school-age children over 10 years of age [12,13,14]. Studies that have evaluated preschoolers have small sample sizes and do not include GMC in their analysis variables [15,16,17].

Given the low levels of PF and the high indicators of physical inactivity in children and its prevalence in the development of obesity and comorbidities, it is important and necessary to conduct studies that evaluate the preschool population at an early age, with the aim of providing information that supports decision-making at the national level [18]. Concerned about this issue, several studies have developed research on early childhood, establishing parameters to support strategies that benefit the health of the child population [19,20,21,22].

Stodden et al. (2008) [3] propose a conceptual model with synergistic relationships between MC, physical activity, physical fitness and weight status. Among the various relationships described, it is stated that children with good and high levels of MC would have better PF values, a relationship that becomes reciprocal when they reach adolescence, which would be related to better health, as well as greater participation in physical activity in adulthood. Studies that have examined the relationship between MC scores and total PF suggest a positive relationship between the two [23,24,25,26]. A similar situation has been reported when relating to PF components, where it is supported that MC is positively associated with cardiorespiratory endurance and muscle strength, while the data are uncertain for flexibility [27]. Despite evidence supporting MC, it has been reported that children worldwide have low levels of MC [28,29], a situation that will lead children to be physically inactive and therefore develop low levels of PF [3].

It is essential to study the preschool period, which is considered critical for the development of motor skills [30]. During the first years of life, the brain and central nervous system grow based on new neural connections generated by daily stimuli [6]. In addition, reflexes and GMC, which form the basis of advanced motor behaviour [30], are developed. Research in this population has described MC levels, the relationship with weight status, the improvement with the intervention or the relationship between real and perceived MC [22,31,32,33,34]. It has even been reported that gender differences in MC are already evident at this age [35]. With respect to studies on MC and PF, the establishment of reference values is known [36,37,38,39,40,41], but there are a few studies in the international context that have investigated the association between GMC and PF in preschool children, among which two report an association [42,43], while another found no association [44]. Given that no studies were found in Chilean children that address the association between the two components, generating new information could contribute to a better understanding of the topic, highlighting the importance of early assessment and stimulating the design of programs aimed at improving MC and PF in the first years of life. For this reason, the aim of this research is to analyse the association between gross motor competence and physical fitness, controlling for possible confounding factors. The hypothesis of the study is that there is a positive association between gross motor competence and physical fitness when controlling for confounding factors.

## 2. Materials and Methods

### 2.1. Study Design and Participants

Observational, relational and cross-sectional studies using a quantitative approach. The sample was non-probability and convenience. All children in the prekindergarten and kindergarten classes of a subsidised educational institution in the municipality of Temuco, Araucanía Region, Chile, were invited to participate. This institution was chosen given the ease of access to collect data and mainly because it is an educational institution that has many students with several courses per grade, which guarantees having a sample with boys and girls in the age range targeted by this study.

The inclusion criteria were age between 4 and 6 years, having completed the PF and GMC tests in their entirety, parental consent to allow their child to participate in the study and consent of the child when asked to participate. Children were excluded if they had a physical, mental and/or chronic illness that prevented them from participating in the assessments, which were informed by the educational establishments and/or by the parents. The PF and GMC tests were scored by graduate sports teachers who were trained prior to the research to ensure that instructions and procedures were understood and followed, thus ensuring standardisation. The final sample included 144 children (63 boys and 81 girls) (the data recruitment process can be consulted in Figure S1 of the Supplementary Materials). Data were collected between May and June 2019. Prior to the evaluation, the principal investigator sent the informed consent forms to the students, which had to be signed and submitted as a requirement for participation. The consents were given on the day of the measurements.

All physical assessments were conducted by the principal investigator in a 90 min session during school hours in the school’s gymnasium and in collaboration with a group of external physical education teachers who received training to ensure standardization, which consisted of a first part where they were given all the theoretical information about the tests to be used. The second part consisted of a practical part where the tests were executed to analyse their correct implementation and the most common errors. In a third stage, a group of children aged 4 to 6 years was invited and evaluated. The fourth and final stage consisted of going to an educational establishment and evaluating children from 4 to 6 years old. After this entire process, the evaluations were carried out in the included educational establishments. The schoolteachers always supervised the evaluations.

The Universidad Autónoma de Chile, through its Scientific Ethics Committee, approved the development of this research (No. 11–19). In addition, all procedures were carried out in accordance with the Declaration of Helsinki.

### 2.2. Instruments

Gross motor competence: it was evaluated using the Movement Assessment Battery, second edition (MABC-2), in its validated version in Spanish [45,46], using the motor tasks that evaluate gross motor competence, from the version for the range from 3 to 6 years, which are: catch and aim (2 tests), static and dynamic balance (3 tests).

The MABC-2 instrument includes more tests than those incorporated in this study that evaluate fine MC, which together provide a total score that indicates the level of motor development; however, in this study, they were not evaluated since in the included educational establishments The necessary infrastructure conditions were not there to be applied. The calculation of the GMC level was carried out in the following way: the raw value delivered by each of the evaluated tests was converted into a normative value as proposed in the MABC-2 manual. The sum of the results of each test was used to calculate the gross motor skills. The higher the score obtained, the better the performance in gross motor skills [45]. As the MABC-2 battery does not establish categories when assessing gross motor skills alone, and because we want to compare between categories, participants were divided into quartiles: low (first quartile), medium (second and third quartiles) and high (fourth quartile).

Physical fitness: This was assessed using some tests that are part of the battery for assessing FITness in PREschool (PREFIT) [47]. Cardiorespiratory fitness was assessed using the 20 m shuttle run test. The last stride achieved was recorded. Lower-body muscular strength was assessed using the long jump test. The best of three attempts was recorded in centimetres. Speed/agility was measured using the 4 × 10 m sprint test. The worst of two attempts was recorded in seconds. The test sequence proposed in the battery manual was followed, as well as having the children perform them wearing sports clothing and shoes. At the time of carrying out the tests, messages of encouragement and motivation were given to those evaluated to help them achieve their maximum performance, all of this because of the age of the participants and because it was proposed by the authors of the PREFIT battery.

Anthropometric measurements: Weight was measured using a digital scale (Omrom, Kyoto, Japan) with an accuracy of 0.1 kg. Height was measured with a stadiometer (Seca 222), with an accuracy of 0.1 cm. Both were measured twice. The mean value was used to calculate the body mass index (BMI).

### 2.3. Statistical Analysis

The normality of variables was assessed using the Kolmogorov–Smirnov test, whose values showed a normal distribution, choosing parametric tests for subsequent analyses. A Student’s *t*-test was used to determine gender differences. To examine associations between the components of GMC and PF, the Pearson correlation coefficient was used, by gender and total sample, controlled for age. Values were considered trivial, small, moderate or large if they were in the ranges <0.1, 0.1–0.29, 0.3–0.49 and ≥0.5, respectively [48].

To test for differences between PF components by coarse GMC categories, analysis of covariance (ANCOVA) models were used, controlling for age (model 1) and then including BMI as a covariate (model 2). For these effects, the assumptions of linearity, homogeneity of the variance in each group and independence between the co-variable and the dependent variable were checked. The Bonferroni post hoc test was used for multiple comparisons (between groups). For ANCOVA, partial eta squared (η^2^_p_) was calculated, indicating a small (0.01), medium (0.06) or large (0.14) effect size [48]. Data were analysed using IBM statistical software, version 29. The significance level was set at *p* < 0.05.

## 3. Results

The main characteristics of the sample are presented in Table 1, by total values and by gender. For anthropometric measures, boys have significantly higher scores for height, while girls have higher scores for weight and BMI, with no significant differences.

For physical fitness, boys have better scores for lower body strength and speed/agility, with significant differences only for the former. Girls have better values for cardiorespiratory fitness, but the differences are not significant.

For GMC, girls had higher values, but these differences were not significant. The values of each test to obtain GMC can be seen in Figure S2 of the Supplementary Materials.

The partial correlation coefficients for GMC and BMI with the different components of PF, controlled for age, total sample and gender, are presented in Table 2. Regarding GMC, it correlated positively and weakly with the total sample, girls and boys, for lower body strength; positively and moderately with the total sample and positively and weakly with girls and boys for agility/speed; positively and weakly with the total sample and boys, and positively and moderately with girls for cardiorespiratory fitness and finally, it correlated negatively and weakly with the total sample for BMI. As for what was found for BMI, there was a negative and small correlation with the total sample, girls and boys, for cardiorespiratory fitness.

The results of the mean differences in the PF tests as a function of GMC categories, controlled by age (model 1) and by age and BMI (model 2), for the total sample and by gender, are presented in Table 3. They can also be consulted in the Supplementary Material in Figure S3. In both models, those in the high GMC category have significantly higher results in the lower body muscular strength and cardiorespiratory fitness components for the total sample, girls and boys. On the other hand, those in the high GMC category have significantly higher scores in the lower body muscular strength and cardiovascular fitness components for the total sample, girls and boys. In terms of effect size, large values were found for the lower body strength component in model 1 for boys and in model 2 for the total sample of girls and boys. In all other cases, the effect size was small.

## 4. Discussion

The aim of this research was to analyse the association between GMC and PF, controlling for possible confounding factors. The main findings of this study indicate that girls and boys have a low association between the components of physical fitness, lower body muscle strength and cardiorespiratory fitness and GMC. In addition, girls and boys with higher GMC scores have better levels of lower body muscle strength and cardiorespiratory fitness, mostly with a small effect size and after controlling for potential confounders, which is an expected outcome and which serves as evidence to highlight the importance of developing GMC in preschoolers.

The descriptive values of the anthropometric characteristics of the evaluated sample are similar to those reported in Chilean children of the same age [16,17,49]. However, BMI values are higher than those reported for children of the same age in Europe, Africa and Asia [50,51,52,53,54], a situation that is in line with what has been reported for more than a decade in children and adolescents, not only in Chile but also throughout Latin America [55,56,57,58], which is worrisome because there is ample evidence that this condition is maintained in adulthood [59], increasing the risk of contracting noncommunicable diseases [60,61] and premature death [62].

The descriptive values obtained in PF are similar to those of the preschool population in Chile [14,15,16,17], but lower than those reported in other countries [42,43,44]. This situation could be explained by the long length of the Chilean school day, which leads to low energy expenditure [63,64], low levels of physical activity [18], high levels of overweight and obesity [65], as a result of poor nutrition [17,65,66]. Important aspects to highlight in the evaluation of PF in this population are, first, the scarcity of research at the national level and, second, the use of different tests and/or protocols, which make it difficult to make comparisons or establish trends over time. For this reason, it is essential to establish a PF assessment battery to be used with Chilean preschool children. In this sense, we believe that the PREFIT battery meets all the conditions to be used with preschoolers, so we encourage further research using this instrument.

Regarding the GMC, girls have higher average scores than boys, a situation that has been reported in studies with children of the same age when analysing the total values [67,68], since studies that have compared the tests that make up the batteries used, report that girls mostly have better values in static and dynamic equilibrium, while children have them in aim and caught [69,70]. A possible explanation for this situation could be the activities they carry out, where girls prefer those with an expressive-rhythmic-motor component in which coordination predominates (dance, exercises with music and jumping rope), while boys mostly perform activities in which strength predominates (soccer, fights and pushing) [71,72]. Added to this are the gender stereotypes established by society in childhood, where it is decided from an early age what it means to be a girl or a boy and what each one should do [73].

Regarding the associations between GMC and PF, as far as we know, there are no studies that have analysed them in the Chilean preschool population, while at the international level, there are a few studies that have reported them, all of them with different results [42,43,44]. The results of this study indicate a positive association between GMC and the components of lower body muscle strength, speed/agility and cardiorespiratory fitness, results similar to those reported in older children, which would suggest that the sample evaluated would lay the foundations for an adequate development of the basic skills necessary for good participation in motor activities [74,75], as well as a greater probability of obtaining health benefits [27,76], a situation that is relevant given the high rates of overweight, obesity and low levels of physical activity in children in Chile [18,65].

In the models analysed, one adjusted for age and the other for age and BMI, no differences in associations were observed. We believe this is because, at these ages, BMI is not able to accurately reflect the differences between different components of the body [77]. Although no differences in the models are reported in this study when BMI is included, it is of utmost importance to monitor this index at older ages, since there is robust evidence that establishes it as an important indicator of health in children and adolescents [59].

Regarding the correlations between BMI and the different components of physical fitness in boys and girls, studies deliver similar results, showing that higher levels of GMC correlate with better fitness in both boys and girls [20,43,52,78]. However, the results of the correlations between GMC and BMI were lower compared to a study conducted in South Africa [43], which only found an inverse correlation with cardiorespiratory fitness (r= −0.127), a situation that could be due to factors such as the level of physical activity and sociodemographic, environmental and nutritional factors, among others.

Another of our findings was that those in the high GMC category in both models had significantly higher scores for lower body muscle strength and cardiorespiratory fitness in the total sample, girls and boys, and for the speed/agility component in the total sample and boys. These results support the conceptual model developed by Stodden et al. (2008) [3], which proposes that children with good and high levels of MC would have better PF scores, which would contribute to better health and greater participation in physical activity in adolescence and adulthood [79].

Although the results found in this research were expected, since the evidence indicated that those with better values of GMC also had better values in physical fitness, they have value in practice because they allow ratifying, this time in Chilean children, the benefits of working from an early age on the variables studied, which serves as evidence for educational establishments, as well as for families, incorporate the work of these variables.

Our study is limited by its cross-sectional design, which does not allow us to determine cause and effect, so longitudinal studies are needed to draw firm conclusions. Using a non-representative sample also limits the findings of our study. The strengths of this research lie in working with a population in which the variables of interest have been little studied, and this research is a contribution that may be useful for other researchers or interventions. 

Studies that include the measurement of fine MC together with GMC are recommended to have a global view of the relationship between MC and PF. It is also recommended to include variables such as perceived MC and physical activity. 

## 5. Conclusions

The results of our study reported differences in GMC and PF when compared by sex, but these were not significant. Low associations were found between GMC and PF components. In addition to controlling for confounding variables such as age and BMI, people with higher GMC scores have better levels of lower body muscle strength, speed/agility and cardiorespiratory fitness. Interventions developed for preschool children, both in educational settings and in government programs, should consider the work of the GMC to improve PF components. Although the inclusion of BMI as a confounding variable did not alter the associations found in the sample studied, it is important to monitor this indicator due to its relevance to the health of children and adolescents.

## Figures and Tables

**Table 1:** Sample characteristics.

	Total (*n* = 144)	Girls (*n* = 81)	Boys (*n* = 63)	*p*	*d*	*CI*	
Age (years)	5.32 ± 0.77	5.27 ± 0.77	5.38 ± 0.77	0.401	−0.141	−0.366	0.147
Height (cm)	115.57 ± 9.37	114.09 ± 7.44	117.48 ± 11.16	**0.031**	−0.366	−6.462	−0.318
Weight (kg)	23.62 ± 5.24	23.63 ± 5.30	23.60 ± 5.21	0.976	0.005	−1.719	1.774
BMI (kg/m^2^)	17.63 ± 2.67	17.98 ± 2.52	17.20 ± 2.82	0.082	0.294	−0.100	1.661
Lower-body muscular strength (cm)	80.23 ± 21.88	76.63 ± 19.56	84.84 ± 23.91	**0.025**	−0.381	−15.375	−1.049
Speed/agility ^¥^ (s)	17.15 ± 1.82	17.37 ± 2.04	16.87 ± 1.46	0.105	0.074	−0.105	1.096
Cardiorespiratory fitness ^‡^ (stage)	1.74 ± 0.86	1.75 ± 0.90	1.71 ± 0.80	0.802	0.042	−0.251	0.324
Gross motor competence (MABC-2)	56.91 ± 10.83	58.12 ± 10.09	55.35 ± 11.61	0.128	0.257	−0.805	6.354

The data are presented as means and standard deviations. ^¥^ Less time (in seconds) indicates better fitness level, ^‡^ 1 stage = 1 min. The values in bold indicate a statistical significance for *p* < 0.05.

**Table 2:** Correlations between GMC and BMI as a function of health status.

		Lower-Body Muscular Strength	Speed/Agility	20 m Shuttle Run Test	BMI	GMC
GMC	Total	0.112 **	0.367 **	0.164 **	−0.151 *	
	Girls	0.108 **	0.198 **	0.333 **	−0.115	
	Boys	0.209 **	0.276 **	0.155 **	−0.147	
BMI	Total	−0.116	−0.119	−0.127 **		−0.151 *
	Girls	−0.122	−0.102	−0.128 **		−0.115
	Boys	−0.163	−0.138	−0.138 **		−0.147

Values of <0.1, 0.1–0.29, 0.3–0.49 and ≥0.5 were considered trivial, small, moderate or large, respectively (Cohen 1988 [48]). * *p* < 0.05; ** *p* < 0.01.

**Table 3:** Mean differences (SE) in physical fitness by gross motor ability categories, controlling for confounders, by gender.

			Gross Motor Competence										
		Model 1						Model 2					
		Low (n = 39) G22/B17	Medium (n = 73) G41/B32	High (n = 32) G18/B14	*p*	η^2^_p_	β	Low (n = 39) G22/B17	Medium (n = 73) G41/B32	High (n = 32) G18/B14	*p*	η^2^_p_	β
Lower-body muscular strength (cm)	Total	68.02 ± 23.73	82.48 ± 18.51	89.94 ± 20.64	**0.00**	0.15	0.99	67.84 ± 24.01	82.48 ± 18.5	89.29 ± 20.65	**0.00**	0.14	0.98
	Girls	67.02 ± 17.05	81.45 ± 15.85	87.95 ± 15.87	**0.00**	0.13	0.74	66.79 ± 23.43	81.52 ± 18.97	87.89 ± 20.91	**0.00**	0.17	0.75
	Boys	69.09 ± 26.03	83.52 ± 19.94	91.85 ± 23.34	**0.00**	0.25	0.98	68.89 ± 24.87	83.44 ± 18.34	90.69 ± 20.03	**0.00**	0.21	0.96
Speed/ agility (s)	Total	17.72 ± 2.13	17.11 ± 1.54	16.54 ± 1.84	**0.01**	0.05	0.75	17.73 ± 2.15	17.11 ± 1.53	16.56 ± 1.86	**0.01**	0.05	0.70
	Girls	17.98 ± 2.54	17.45 ± 1.87	16.99 ± 2.97	0.34			18.62 ± 1.94	17.82 ± 1.98	17.12 ± 1.66	0.29		
	Boys	16.51 ± 1.98	16.22 ± 2.42	16.09 ± 2.45	**0.00**	0.12	0.71	16.84 ± 2.67	16.42 ± 2.34	16.02 ± 0.86	**0.00**	0.13	0.60
20 m shuttle run test (stage)	Total	1.46 ± 0.76	1.73 ± 0.92	2.08 ± 0.69	**0.01**	0.07	0.84	1.46 ± 0.76	1.73 ± 0.91	2.07 ± 0.68	**0.00**	0.07	0.77
	Girls	1.26 ± 0.45	1.55 ± 0.77	1.82 ± 0.67	0.00	**0.15**	0.92	1.01 ± 0.34	1.25 ± 0.87	1.59 ± 0.12	**0.00**	0.08	0.95
	Boys	1.66 ± 1.23	1.91 ± 0.98	2.35 ± 0.45	**0.00**	0.09	0.73	1.91 ± 0.94	2.21 ± 1.18	2.55 ± 0.56	**0.00**	0.09	0.69

Model 1: controlling for age. Model 2: controlling for age and BMI. The values in bold indicate a statistical significance of *p* < 0.05.

## Data Availability

The data presented in this study are available on request from the corresponding author. The data are not publicly available due to ethical standards.

## Supplementary Materials

The following supporting information can be downloaded at: https://www.mdpi.com/article/10.3390/children11050561/s1, Figure S1. Flow diagram of participant recruitment and exclusion with reason. Figure S2. Results obtained in tests to obtain gross motor competence. Figure S3. Mean differences in physical fitness by gross motor competence categories, controlling for confounders, by gender.
