# Supplementary material for: Association between Gross Motor Competence and Physical Fitness in Chilean Children Aged 4 to 6 Years
Source: Children (Basel). 2024 May 8;11(5):561. doi: 10.3390/children11050561 (PMC11119627; doi:10.3390/children11050561)
Supplement: Supplementary file 1 [file children-11-00561-s001.zip › children-2961531-supplementary.pdf]

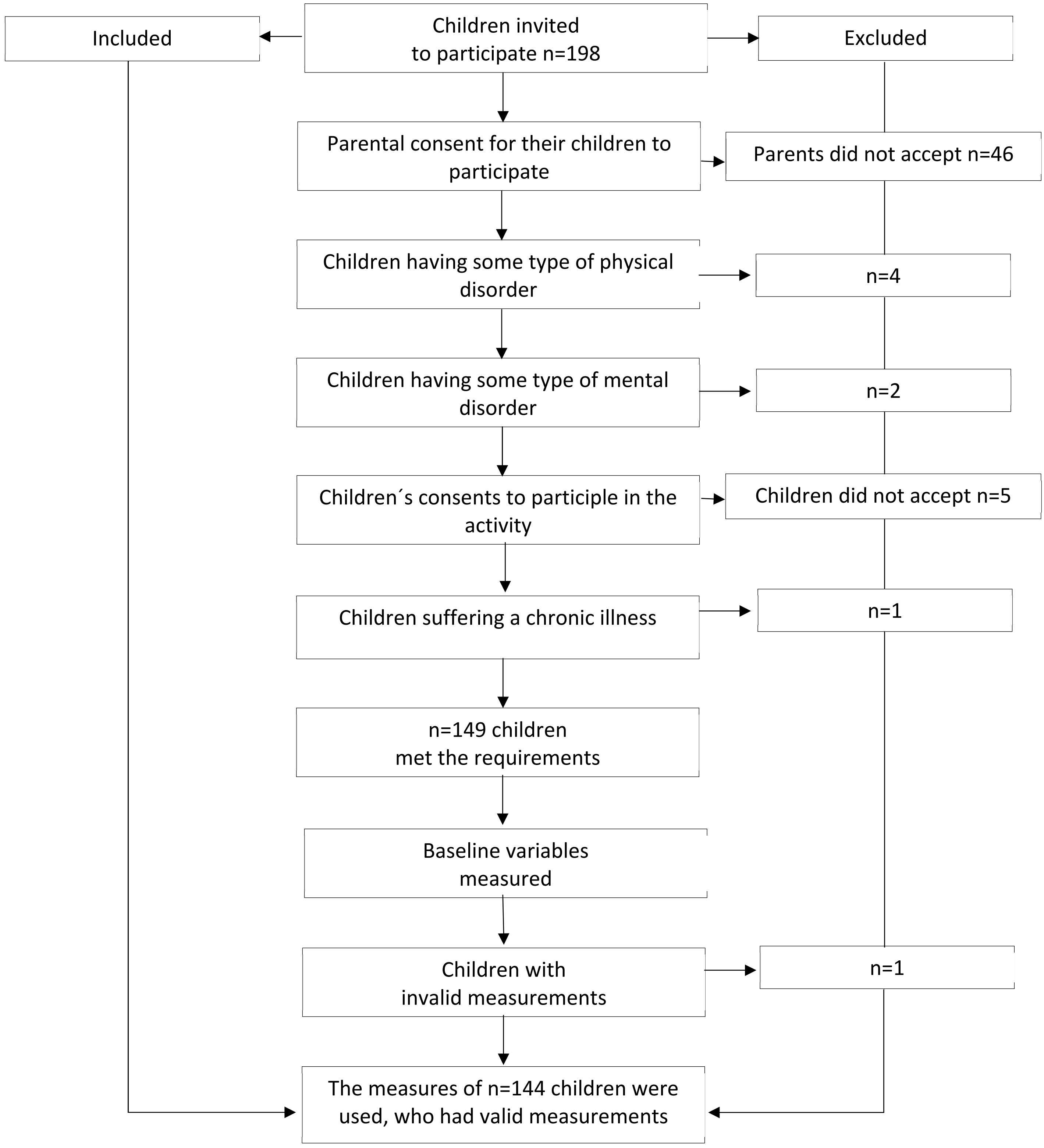

Figure S1. Flow diagram of participant recruitment and exclusion with reason

Subject	Gender	Age	Catching	Aiming	Right balace	Left balance	Tiptoes	Floor mat
Subject 1	0	5	1	1	3	14	0	4
Subject 2	0	5	0	0	3	1	15	5
Subject 3	0	5	5	2	4	6	12	4
Subject 4	0	6	3	5	5	6	15	4
Subject 5	0	6	8	3	3	3	15	4
Subject 6	0	5	3	2	13	23	5	5
Subject 7	0	5	7	4	4	3	10	4
Subject 8	0	4	3	2	5	7	8	5
Subject 9	0	5	5	5	8	17	5	5
Subject 10	0	4	1	1	5	3	15	4
Subject 11	0	4	5	4	3	6	0	5
Subject 12	0	5	4	2	8	8	15	5
Subject 13	0	5	5	1	5	11	15	5
Subject 14	0	4	4	3	3	2	13	5
Subject 15	0	6	10	4	4	3	15	5
Subject 16	0	6	7	3	15	10	15	5
Subject 17	0	6	7	2	15	14	15	5
Subject 18	0	5	6	2	14	8	15	5
Subject 19	0	6	7	2	21	15	13	5
Subject 20	0	5	9	1	6	3	15	5
Subject 21	0	6	7	5	13	5	15	5
Subject 22	0	6	8	4	15	10	15	5
Subject 23	0	4	6	5	3	2	15	4
Subject 24	0	6	5	4	13	26	15	5
Subject 25	0	5	10	2	7	6	15	5
Subject 26	0	5	9	1	10	8	15	5
Subject 27	0	6	10	3	19	9	15	5
Subject 28	0	5	10	3	8	9	15	5
Subject 29	0	5	5	8	16	9	15	5
Subject 30	0	5	9	2	14	13	15	5
Subject 31	0	5	5	6	17	15	15	5
Subject 32	0	6	10	5	12	17	15	5
Subject 33	0	6	8	6	15	19	15	5
Subject 34	0	5	9	2	11	30	15	4
Subject 35	0	5	9	6	10	15	11	5
Subject 36	0	6	8	6	21	15	15	5
Subject 37	0	6	8	6	20	17	15	5
Subject 38	0	4	5	1	30	6	15	5
Subject 39	0	4	6	5	13	17	15	3
Subject 40	0	4	5	4	21	6	15	5
Subject 41	0	4	6	1	19	11	15	5
Subject 42	0	6	7	5	30	30	15	4
Subject 43	0	6	10	7	12	19	15	5
Subject 44	0	6	8	3	30	30	15	5
Subject 45	0	4	9	5	4	3	15	5
Subject 46	0	4	7	3	20	3	15	5
Subject 47	0	4	7	2	3	22	15	5
Subject 48	0	5	10	6	10	8	15	5
Subject 49	0	5	5	8	10	30	15	5
Subject 50	0	6	10	5	30	10	15	5
Subject 51	0	6	9	2	30	30	15	5
Subject 52	0	6	6	5	30	30	15	5
Subject 53	0	6	8	4	30	30	15	5
Subject 54	0	5	10	2	15	30	15	5
Subject 55	0	6	6	9	19	30	15	5
Subject 56	0	6	10	3	30	30	15	5
Subject 57	0	6	5	7	30	30	15	5
Subject 58	0	6	10	4	26	30	15	5
Subject 59	0	6	9	4	30	30	15	5
Subject 60	0	6	8	5	30	30	15	5
Subject 61	0	4	8	2	30	7	15	5
Subject 62	0	5	10	3	14	30	15	5
Subject 63	0	5	10	3	15	30	15	5
Subject 64	0	6	10	4	30	30	15	5
Subject 65	0	6	10	4	30	30	15	5
Subject 66	0	6	10	4	30	30	15	5
Subject 67	0	6	10	4	30	30	15	5
Subject 68	0	6	10	5	30	25	15	5
Subject 69	0	6	9	5	30	30	15	5
Subject 70	0	5	8	3	30	30	15	5
Subject 71	0	6	10	5	30	30	15	5
Subject 72	0	6	10	5	30	30	15	5
Subject 73	0	5	9	5	22	27	15	5
Subject 74	0	6	10	6	30	30	15	5
Subject 75	0	5	8	5	30	30	15	5
Subject 76	0	6	10	8	30	22	15	5
Subject 77	0	4	7	3	24	21	15	5
Subject 78	0	4	8	4	19	20	15	5
Subject 79	0	6	10	9	30	30	15	5
Subject 80	0	5	9	7	30	30	15	5
Subject 81	0	4	9	4	30	30	15	5
Subject 82	1	4	1	1	1	1	0	3
Subject 83	1	6	9	3	6	2	0	1
Subject 84	1	5	5	3	12	6	4	3
Subject 85	1	6	0	4	2	5	15	4
Subject 86	1	6	8	2	6	3	6	5
Subject 87	1	6	9	0	14	4	4	5
Subject 88	1	6	8	4	6	13	5	5
Subject 89	1	5	7	4	4	3	10	4
Subject 90	1	6	9	2	8	16	15	3
Subject 91	1	6	10	2	2	5	15	5
Subject 92	1	4	5	4	16	7	6	3
Subject 93	1	6	7	6	3	9	15	4
Subject 94	1	6	8	4	19	20	2	5
Subject 95	1	5	10	5	6	5	0	5
Subject 96	1	6	6	6	9	20	15	3
Subject 97	1	6	10	1	20	5	14	4
Subject 98	1	6	6	5	5	9	15	5
Subject 99	1	5	7	6	16	7	6	5
Subject 100	1	4	7	7	5	2	12	3
Subject 101	1	4	4	1	7	10	15	4
Subject 102	1	5	4	7	10	9	15	5
Subject 103	1	6	9	4	13	9	15	5
Subject 104	1	6	10	4	8	13	15	5
Subject 105	1	5	9	3	9	8	15	4
Subject 106	1	5	3	8	8	19	15	5
Subject 107	1	6	7	3	22	30	15	4
Subject 108	1	4	8	4	10	15	10	3
Subject 109	1	4	8	3	5	9	8	5
Subject 110	1	5	10	4	6	14	10	5
Subject 111	1	6	10	6	21	11	15	3
Subject 112	1	6	10	6	14	18	10	5
Subject 113	1	6	10	5	12	12	15	5
Subject 114	1	4	10	2	3	4	13	5
Subject 115	1	6	10	7	3	15	15	5
Subject 116	1	6	10	5	9	20	15	5
Subject 117	1	5	10	7	2	2	15	5
Subject 118	1	6	9	6	4	30	15	5
Subject 119	1	6	6	8	23	30	15	3
Subject 120	1	6	9	3	30	30	15	4
Subject 121	1	6	10	4	15	30	15	5
Subject 122	1	4	9	7	8	6	15	3
Subject 123	1	5	10	5	13	30	8	5
Subject 124	1	5	10	4	5	20	15	5
Subject 125	1	6	9	5	30	8	15	5
Subject 126	1	6	9	2	30	26	15	5
Subject 127	1	6	10	3	20	30	15	5
Subject 128	1	5	9	4	25	5	15	5
Subject 129	1	5	10	4	18	11	15	5
Subject 130	1	6	10	4	30	19	15	5
Subject 131	1	6	10	6	16	25	15	5
Subject 132	1	4	8	3	16	16	15	4
Subject 133	1	6	9	6	30	30	15	4
Subject 134	1	6	10	4	30	30	15	5
Subject 135	1	6	9	6	30	30	15	5
Subject 136	1	6	9	7	30	30	15	5
Subject 137	1	4	7	3	15	30	15	5
Subject 138	1	5	9	4	30	30	15	5
Subject 139	1	6	10	7	30	28	15	5
Subject 140	1	6	8	9	30	30	15	5
Subject 141	1	5	10	4	30	30	15	5
Subject 142	1	4	9	2	19	27	15	5
Subject 143	1	5	10	6	30	30	15	5
Subject 144	1	5	10	6	30	30	15	5

Figure S2. Results obtained in tests to obtain gross motor competence.

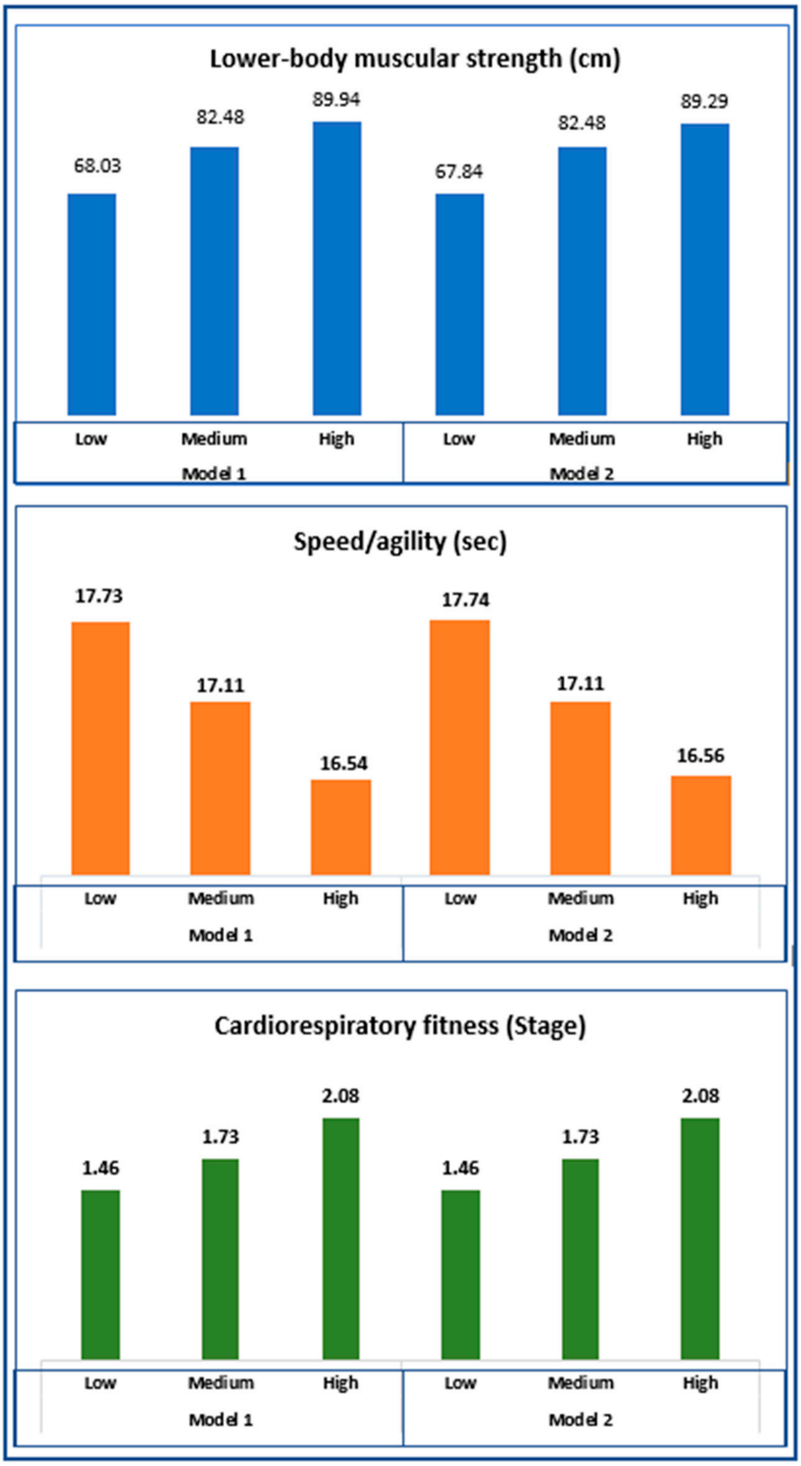

Figure S3. Mean differences in physical fitness by gross motor competence categories, controlling for confounders, by gender.
